# Does Job Role Matter? Food Safety Knowledge and Training Effectiveness Among Food Handlers in Collective Catering

**DOI:** 10.3390/foods15132298

**Published:** 2026-06-26

**Authors:** Giovanni Centonze, Carlo Di Pietrantonj, Elisa Allocco, Elena Kyoko Canova, Matteo Papurello, Elena Lenta, Manuela Alessio, Antonella Beccafico, Federica Leone, Noemi Farulla, Giorgio Boffa, Davide Marcellino, Sabrina Contini, Giulia Picciotto, Paolo Borello, Giuseppe Calabretta, Pietro Maimone, Laura Marinaro

**Affiliations:** 1 Food Safety and Nutrition Service (S.I.A.N.), Department of Prevention, Azienda Sanitaria Locale ASL CN2, 12051 Alba-Bra, Italy; 2 Epidemiology, Health Promotion and Coordination of Prevention Activities, Department of Prevention, Azienda Sanitaria Locale ASL CN2, 12051 Alba-Bra, Italy; 3 Projects, Research and Innovation, Azienda Sanitaria Locale ASL CN2, 12051 Alba-Bra, Italy; 4 Directorate of Health Professions, Azienda Sanitaria Locale ASL CN2, 12051 Alba-Bra, Italy; 5 Prevention and Safety Unit (SPeSAL), Department of Prevention, Azienda Sanitaria Locale ASL CN2, 12051 Alba-Bra, Italy

**Keywords:** food safety knowledge, HACCP training, collective catering, food handlers, pre–post training session, residential care facilities, school cafeterias

## Abstract

Food safety training is a cornerstone of foodborne disease prevention in collective catering, particularly in settings serving vulnerable populations. This study aimed to assess baseline food safety knowledge and evaluate the effectiveness of a food safety training session among food handlers employed in school cafeterias and residential care facilities (RSAs) collective catering. A pre–post design was applied to 168 participants who completed a structured knowledge questionnaire before and after training. At baseline, only 31% of participants achieved a passing score. Knowledge levels were significantly associated with primary job role (*p* < 0.001): food preparers and managers were more likely to pass compared with food service workers involved mainly in meal distribution. In multivariate analysis, both job role and catering setting remained independently associated with test performance. Following the training session, the proportion of participants passing the test increased to 74% (*p* < 0.001), and differences between professional categories were reduced. These findings indicate that food safety knowledge in collective catering could vary according to occupational role and organizational context, but can be improved through training. Role-tailored, HACCP-based educational programs could be essential to strengthen compliance and protect vulnerable populations in institutional catering settings.

## 1. Introduction

Foodborne diseases (FBDs) continue to represent a major public health challenge worldwide, despite advances in food safety regulations and control systems. The World Health Organization estimates that 600 million people worldwide contract a foodborne illness, with a disproportionate burden among vulnerable populations such as children, the elderly, and individuals with compromised health conditions [1]. In these settings, collective catering services such as school cafeterias and residential care facilities (RSAs) play a crucial role, as they provide meals to high-risk population groups and are often characterized by large-scale food production and unique challenges due to their organizational structures and high food turnover. In accordance with the current legislation, to ensure food safety, catering services are required to comply with general hygiene regulations and to implement permanent procedures based on Hazard Analysis and Critical Control Point (HACCP) principles aimed at identifying hazards and preventing risks throughout the food production process [2].

Despite these regulatory frameworks, human factors remain a major contributor to food safety failures [3,4,5]. Food handlers are directly responsible for food preparation, storage, and service, and their knowledge, attitudes, and practices are widely recognized as key determinants of food safety outcomes. Common errors in foodservice settings such as inadequate personal hygiene, cross-contamination, insufficient equipment sanitation, and poor temperature management significantly heighten the risk of FBDs, impairing the health of consumers [6,7,8]. Improving food safety requires continuous training of food handlers, strengthened HACCP procedures, and increased awareness of high hygiene standards within collective catering environments. In these settings, several studies have shown that food safety training generally improves knowledge levels among food handlers: the assessment of food handlers’ knowledge, attitudes, and practices represents a valuable tool for evaluating training effectiveness and for prioritizing actions in the planning of food safety education programs [5]. Moreover, evidence from a systematic review and meta-analysis indicates that food safety training is most effective when it is frequent, context-specific, promotes active learning, enhances risk perception, and takes into account the actual working environment of food handlers [9]. Although food safety training is a legal requirement under European legislation (Regulation (EC) No. 852/2004 [2]), its organization and implementation in Italy are governed by regional regulations, leading to variability in training programs across the country [10]. Furthermore, some regions provided specific guidelines, taking into account European and Italian laws but without specific indications at the regional level [10].

Food safety knowledge can be influenced by several factors, including educational level, work experience, and previous food safety training. However, the role of occupational responsibilities in shaping food safety knowledge has received comparatively limited attention, despite the marked heterogeneity of tasks and responsibilities within collective catering services. Managers, food preparers, and food service workers are involved in different stages of food production and distribution and may therefore be exposed to distinct food safety risks, control measures, and training needs. Investigating these differences may help identify professional groups with greater knowledge gaps and support the development of more targeted educational interventions. Therefore, the aim of this study was to assess food safety knowledge among food handlers working in school cafeterias and residential care facility catering services, with a specific focus on occupational role, and to evaluate the effectiveness of a food safety training session in reducing knowledge gaps across professional categories.

## 2. Materials and Methods

### 2.1. Study Design and Setting

This is an observational pre–post study conducted among food handlers employed in collective catering services within the competence area of the Azienda Sanitaria Locale CN2-Local Health Authority (ASL CN2)—Alba and Bra, Italy. An in-depth standardized food safety training course was offered to food handlers working in school cafeterias and RSA collective catering, and participation was voluntary. Specifically, the course was advertised to all companies operating within the institutional competence area; registration was open and voluntary, and no individual or organization was required to participate. The training program was delivered in four separate editions organized by ASL CN2 from December 2023 to November 2025. Each participant attended one single course edition only, and all four editions were identical in content and structure. Participants registered independently for the course and included food preparers, food service workers (involved exclusively in meal distribution), and personnel with managerial responsibilities. Each course edition was conducted on a single day. The training was designed as a concise yet intensive three-hour session, focusing on essential food safety principles relevant to the daily tasks of collective catering personnel. Before the start of the course, participants completed a structured pre-training questionnaire designed to collect socio-demographic information and to assess baseline knowledge on key food safety topics. The completed questionnaires were collected prior to the training session. At the end of the training, the same questionnaire was administered to evaluate knowledge acquisition and training effectiveness. To minimize response bias, participants were not allowed to review their pre-training responses while completing the post-training questionnaire.

### 2.2. Food Safety Training Course

A 3 h food safety training course was offered to food handlers working in school cafeterias and residential care facility (RSA) collective catering services to strengthen their basic knowledge of food safety, hygiene practices, and food handling in collective catering settings. The training program covered the following core areas of food safety: (A) microbiological hazards and foodborne diseases, including cross-contamination sources and mechanisms, asymptomatic carrier transmission, main foodborne pathogens, and safe cooking temperatures; (B) special dietary needs, including allergen management and vegetarian diets; (C) correct food storage and portioning procedures in collective catering settings; (D) European food safety legislation, with specific reference to the Hygiene Package regulations; and (E) HACCP principles and their application to the food chain, including Critical Control Points monitoring and self-monitoring procedures. The training was delivered through lecturer-led instruction by a multidisciplinary expert panel with an interactive discussion and was designed to be applicable across all professional roles involved in collective catering operations.

### 2.3. Questionnaire and Data Collection

Data were collected using a structured questionnaire administered immediately before (pre-training) and after (post-training) the course. The questionnaire was developed by a multidisciplinary expert panel from the Food Safety and Nutrition Service, comprising physicians (P.B.; P.M.; L.M.), biologists (G.C.), prevention technicians (A.B.; F.L.; N.F.; G.B.; D.M;), and dieticians (E.A.; E.K.C; M.P.; E.L.). Item development was grounded in the training content and current food safety legislation. The questionnaire was self-administered and completed individually. No assistance regarding the content of the questions was provided during questionnaire completion. The pre-training questionnaire included two main sections: (1) Socio-demographic and professional information, such as gender, age, education (less than a high school diploma vs. high school diploma vs. Bachelor’s or Master’s degree), catering setting (school cafeteria vs. RSA), job role, years of experience in catering sector, experience in collective catering, HACCP training course within the previous three years (Yes vs. No) and primary job responsibilities in food handling and food service activities (food preparer vs. food service worker vs. manager); Years of experience in the catering sector and in collective catering services were originally collected using predefined ordinal categories (less than 1 year, 1–3 years, 4–6 years, more than 6 years) and subsequently dichotomized into two groups (≤6 years and >6 years) for regression analyses. (2) A knowledge section consisting of 15 multiple-choice questions, each with a single unambiguously correct answer. The questionnaire assessed factual knowledge directly aligned with the training content and covered key food safety topics, including food contamination, foodborne illnesses, special diets, and HACCP principles. Questions were designed to evaluate knowledge broadly applicable across all professional roles involved in food handling rather than role-specific or highly specialized competencies. A score of ≥11/15 correct answers (>70%) was defined a priori as a pragmatic threshold to identify participants demonstrating an overall adequate level of food safety knowledge. The pre-training questionnaire was completed at the beginning of the course and collected before the start of the training session. The post-training questionnaire, which consisted only of the knowledge section, was administered at the end of the three-hour session to evaluate knowledge acquisition and the effectiveness of the training. Participants were not allowed to review their pre-training responses while completing the post-training questionnaire to minimize response bias. The questionnaire is provided as Supplementary Table S1.

### 2.4. Statistical Analysis

Data were analyzed by descriptive statistics. Associations between socio-demographic and professional characteristics and test performance (passing the knowledge test: No vs. Yes) were assessed using Fisher’s exact test for categorical variables and the Wilcoxon rank-sum test for continuous variables. Similarly, associations between socio-demographic factors and primary job responsibilities (Food Preparers vs. Food Service Workers vs. Managers) were evaluated using Fisher’s exact test for categorical variables and the Kruskal–Wallis test for continuous variables. Univariate and multivariate logistic regression analyses were performed to identify predictors of successful test performance. Odds ratios (OR) with corresponding 95% confidence intervals (CI) are reported. Covariates included in the multivariate model (catering setting, educational level, years of experience, and previous HACCP training attendance) were selected a priori as potential confounders, based on their plausible association with both job role and food safety knowledge. No stepwise variable selection was applied. A graphical data visualization approach was applied to explore response patterns across questionnaire items. For each item, responses were classified into three mutually exclusive categories: correct, incorrect, and don’t know. Trichotomous item responses were represented using the Di Pietrantonj triangular plot which positions each item according to the difference between the proportions of correct and incorrect responses and the proportion of uncertainty [11]. In this graph, each question in the questionnaire is represented by a single point. The items located in the lower-right corner have a higher proportion of correct answers than incorrect ones, whereas those in the lower-left corner have the opposite pattern. Items located near the top corner are characterized by a higher percentage of “don’t know” responses, reflecting greater uncertainty among participants. Consequently, the shift in items toward the lower-right area in the post-training graph can be interpreted as an improvement in knowledge and a reduction in uncertainty. Statistical significance of the difference between the two main response proportions is visually identified when an item lies outside the homogeneity area delimited by the parabola. For further technical details on the analytical pipeline, see Di Pietrantonj [11]. All statistical tests were two-sided, and *p*-values <0.05 were considered statistically significant. Analyses were conducted using the R environment for statistical computing and graphics (R Foundation, Vienna, Austria; Version 4.3.3).

## 3. Results

### 3.1. Characteristics of the Study Population

Overall, 168 food handlers participated in the food safety training courses and were included in the study (Table 1). The study population was predominantly female (82%), with males accounting for 18% of participants. The median age was 50 years (range: 24–64 years). Regarding educational level, most participants had either less than a high school diploma (41%) or a high school diploma (47%), while a smaller proportion held a bachelor’s or master’s degree (13%). For work setting, 93 participants (55%) were employed in school cafeterias, 71 (42%) in RSA collective catering, and 4 (2%) worked in both settings. Regarding primary job responsibilities, most participants were food preparers (n = 90, 55%), followed by food service workers (n = 56, 34%) and personnel with managerial responsibilities (n = 19, 12%). The majority of participants (76%) reported more than six years of experience in the catering sector, and 64% had more than six years of experience specifically in collective catering services. For food safety education, 58% of participants had attended a HACCP training course within the previous three years, while 43% reported no recent HACCP training.

### 3.2. Baseline Item-Level Analysis of Food Safety Knowledge

**Figure 1:**
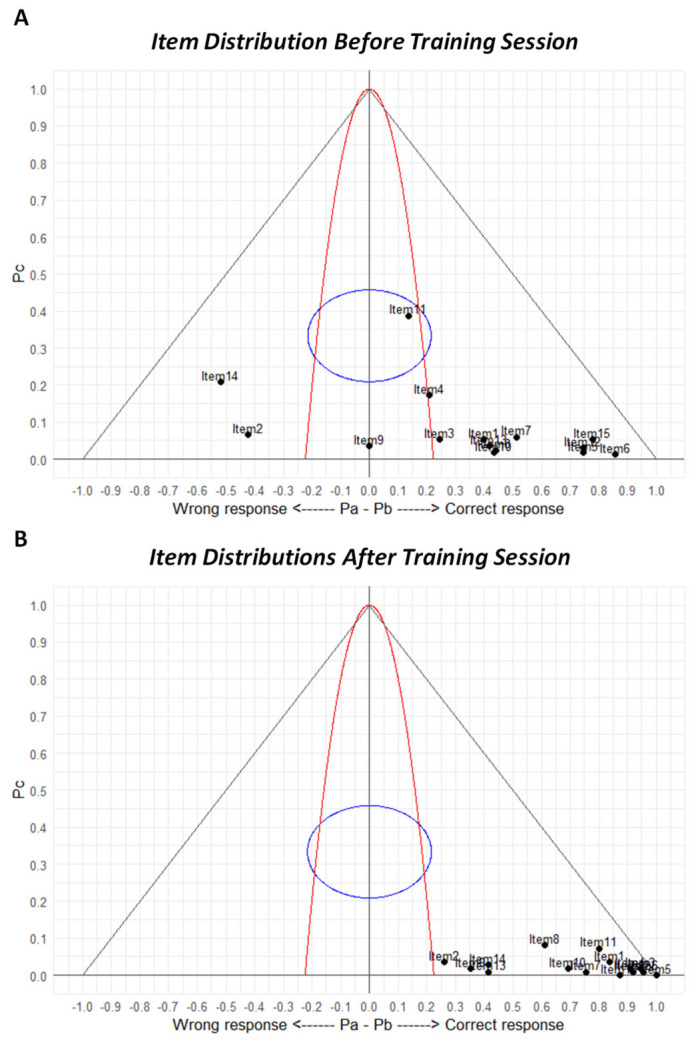
Graphical comparison of pre- (**A**) and post- (**B**) training item distributions. Each point represents a questionnaire item according to the proportions of correct, incorrect, and “don’t know” responses. Items located toward the lower right corner indicate a higher proportion of correct responses, whereas items toward the lower left corner indicate a predominance of incorrect responses. Items near the upper vertex are characterized by greater response uncertainty. The parabola delimits the homogeneity area; points outside this area indicate statistically significant differences between the two main response categories.

Figure 1 provides a graphical summary of response patterns across questionnaire items before and after training. In the pre-training assessment, some items were located in areas characterized by higher proportions of incorrect or uncertain responses. Following the training session, all items shifted toward the lower right region of the plot, indicating an increased proportion of correct responses and a reduction in uncertainty. Specifically, before the training session, incorrect responses predominated for Item 14 (characteristics of official food safety controls) and Item 2 (sensory characteristics of foods acting as vehicles of contamination), reaching areas of statistical significance in the graphical representation. Conversely, no statistically significant predominance of correct or incorrect responses was observed for items addressing regulatory knowledge (food hygiene package regulations—Item 11), portion control practices (portioning of pasta—Item 9), and foodborne pathogens (main causative agents of foodborne diseases in Piemonte—item 4). All remaining items showed a significant predominance of correct responses, suggesting adequate baseline knowledge in those domains.

### 3.3. Baseline Food Safety Knowledge and Associated Factors

**Figure 2:**
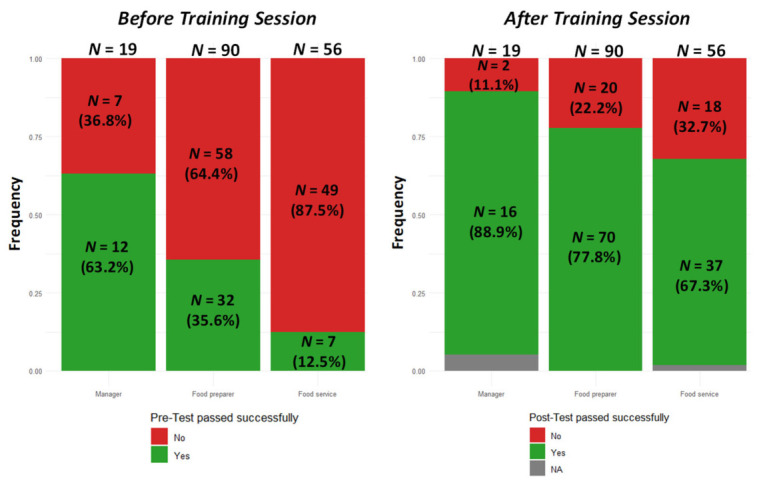
Proportion of participants who passed knowledge test according to primary job responsibilities. NA: not available.

Before the training session, 53 out of 168 food handlers (31%) successfully passed the knowledge test. Baseline test performance was strongly associated with primary job responsibilities (*p* < 0.001). Among food preparers, 36% (n = 32) passed the pre-training test, compared with only 13% (n = 7) of food service workers. In contrast, a higher proportion of participants with managerial responsibilities achieved a passing score (63%, n = 12) (Figure 2). At the item level, several questions showed statistically significant differences in the proportion of correct answers according to job role (Supplementary Table S2). These items addressed key food safety domains, including the absence of sensory alterations in foods acting as vehicles of contamination, the minimum internal temperature required to ensure microbiological safety during cooking, the identification of gluten-free cereals, the applicability of the HACCP system throughout the entire food chain, and the characteristics of official food safety controls. For most of these items, managers and food preparers consistently demonstrated higher levels of correct responses compared with food service workers. In addition, job responsibilities were significantly associated with several socio-demographic and professional characteristics (Supplementary Table S3). Specifically, primary job role was significantly related to educational level (*p* < 0.001), catering setting (*p* = 0.002), overall experience in the catering sector (*p* < 0.001), and attendance at a HACCP training course within the previous three years (*p* < 0.001). Notably, among the 24 participants working in RSA settings who were classified as food service workers according to their food service responsibilities, 15 were health care assistants involved in meal distribution activities, whereas 9 were dedicated catering staff. Univariate logistic regression analysis indicated that food preparers (OR = 3.86, 95% CI 1.65–10.22, *p* = 0.003) and managers (OR = 12.00, 95% CI 3.69–43.60, *p* < 0.001) were significantly more likely than food service workers to pass the knowledge test (Table 2). Additionally, type of canteen (preparation and serving meals vs. delivered meals) was positively associated with successful performance in univariate analysis (OR = 2.60, 95% CI 1.14–6.55, *p* = 0.03). In multivariate logistic regression, after adjusting for catering setting, education, experience, and HACCP course, food preparers remained more likely to pass the test than food service workers (OR = 3.30, 95% CI 1.10–11.10, *p* = 0.04). Furthermore, catering setting (school cafeteria vs. RSA) emerged as a significant predictor in the multivariate model (OR = 2.92, 95% CI 1.16–8.11, *p* = 0.03).

### 3.4. Effect of the Training Session on Food Safety Knowledge

After the training session, correct responses predominated across all items, with most reaching areas of statistical significance in the graphical representation (Figure 1B). Specifically, 125 out of 168 food handlers (74%) successfully passed the knowledge test, representing a 43% increase compared with the pre-test (McNemar *p* < 0.001). The mean number of correct answers increased from 9.4 (SD 2.3) before the training session to 11.9 (SD 2.3) after, with a median score improving from 9 to 12 out of 15 (*p* < 0.001; effect size r = 0.70) (Supplementary Table S4). No significant association was observed between test performance and primary job responsibilities. At the item level, statistically significant differences according to job role were observed for Item 2 (sensory alterations in foods) and Item 14 (characteristics of official food safety controls), with correct responses consistently higher among food preparers and managers compared with food service workers (Supplementary Table S2).

## 4. Discussion

Food safety training is widely recognized as a cornerstone for the prevention of foodborne diseases, particularly in collective catering settings serving vulnerable populations [5,12]. Regulation (EC) No. 852/2004 (Annex II, Chapter XII) requires food business operators to ensure that food handlers receive food hygiene training commensurate with their work activities [2]. Similarly, the Codex Alimentarius General Principles of Food Hygiene emphasize that all personnel involved in food handling should be aware of their responsibilities in preventing food contamination and possess the knowledge and skills necessary to perform their duties hygienically [13]. A recent systematic review and meta-analysis by Young et al. [14] provided strong evidence that training and education programs significantly enhance food safety knowledge among food handlers, supporting the central role of structured learning initiatives in this field. Despite mandatory training requirements in many regulatory frameworks, studies conducted in collective catering and healthcare food service have reported persistent deficiencies in food hygiene knowledge and practices, underscoring the need for rigorous and continuous training programs aimed at improving hazard identification and infection control practices across all staff roles [15,16]. Additionally, within the Italian context, Pattono et al. [10] recently reported substantial regional variability in training approaches, with scheduled periods for refresher courses varying between 2 and 5 years depending on the risk profile, emphasizing that topics such as allergens and gluten are often absent and stressing that education represents a key strategy for fostering a sustainable culture of food safety prevention.

The present study evaluated food safety knowledge and the effectiveness of a food safety training session among food handlers employed in collective catering services. At baseline, food safety knowledge was uneven and frequently insufficient, with only about one-third of participants achieving a passing score, consistent with the current literature. Pre-training knowledge levels differed significantly according to primary job responsibilities, highlighting potential disparities between food preparers, food service workers, and managerial staff. Following the training session, an improvement in immediate post-training knowledge was observed across the entire cohort, with an increase in the proportion of participants passing the post-training assessment and a reduction in knowledge gaps between professional roles.

Baseline differences in food safety knowledge according to primary job responsibilities emerged as a key finding of the present study. Managers and food preparers consistently achieved higher proportions of correct responses, whereas food service workers involved mainly in meal distribution performed markedly worse, particularly on demanding items such as critical cooking temperatures, HACCP applicability, and characteristics of official food safety controls. This occupational gradient mirrors observations from other institutional contexts: in military and hospital catering settings, cooks and nutritionists obtained significantly higher knowledge, attitude, and practice scores than waiters or other service staff, highlighting how job-specific exposure and task responsibilities shape food safety competence [17,18]. In our study, primary job role was also significantly associated with educational level, overall experience in the catering sector, and attendance at a HACCP training course within the previous three years. This suggests that occupational role may function as a proxy for broader structural differences in training exposure and professional background. Similarly, Garayoa et al. [12]. reported that participants with higher educational attainment and longer experience generally performed better on technical items, although relevant gaps persisted in areas such as lethal temperatures for microorganisms and safe holding times. Similarly, Angelillo et al. [15]. also observed that hospital food-service staff with higher education and those working in settings with full HACCP implementation demonstrated significantly greater knowledge regarding pathogen identification and safe storage temperatures, emphasizing the combined influence of formal education, continuing training, and organizational context. These findings indicate that differences in food safety knowledge are influenced by a combination of occupational role, educational background, and prior experience. While general hygiene practices appear to be well internalized across all staff, more complex or technical topics such as temperature control, sensory evaluation limitations, and official control procedures remain weak points, particularly among staff with lower levels of education or less exposure to preparation and technical tasks. These observations suggest the potential value of role-aware and education-tailored training sessions targeting technical knowledge gaps. In practical terms, a modular training approach, in which core food safety principles are delivered to all staff while role-specific modules address the competencies most relevant to each professional category, could represent an effective strategy to optimize training resources and maximize knowledge acquisition across heterogeneous workforces. Food service workers, who demonstrated the lowest baseline knowledge levels, may particularly benefit from targeted modules focusing on microbiological hazards, temperature control, and HACCP monitoring procedures. Given that this study only captures immediate post-training knowledge, continuous and periodic refresher training could remain essential to consolidate learning and ensure that knowledge is maintained and updated over time, particularly in high-turnover settings such as collective catering.

Occupational role remained an independent predictor of successful baseline test performance after adjustment for potential confounders. However, estimates for managerial staff were characterized by wide confidence intervals, indicating uncertainty regarding the precise magnitude of the association, likely reflecting the limited number of participants within this occupational category. In addition to individual professional characteristics, the catering setting remained independently associated with knowledge levels in multivariate analysis. In our sample, the occupational role classification was based on participants’ food responsibilities. Among the 24 RSA participants classified as food service workers according to their role in food distribution and meal service activities, 15 were health care assistants involved in meal distribution activities, whereas 9 were dedicated catering staff. In such contexts, food distribution may therefore be performed by healthcare personnel whose primary professional training may not be specifically focused on food handling. Buccheri et al. [19] reported that nurses and domestic staff are frequently involved in food service operations in hospitals without having received the preliminary and continuous food safety training required for professional food handlers. In their study, substantial proportions of nursing staff involved in food service functions were unaware of critical storage temperatures for ready-to-eat foods and of key etiologic agents and food vehicles associated with foodborne diseases. Comparable knowledge gaps regarding food safety and control in hospital settings have also been described in other studies, emphasizing that the implementation of the HACCP system requires a team-based approach and a shared understanding of monitoring procedures across all staff categories [15,20,21]. These findings suggest that when food handling responsibilities are embedded within broader healthcare roles without clearly defined training requirements, critical knowledge gaps may persist. In institutional settings such as RSAs and hospitals, where meals are provided to elderly, chronically ill, or otherwise vulnerable individuals, these gaps could assume particular relevance. Therefore, when healthcare personnel are involved in food distribution, they effectively act as food handlers and should receive adequate training in accordance with HACCP principles and food safety legislation. Extending food safety education to all staff engaged in food service activities may contribute to ensuring consistent compliance and minimize foodborne risks in facilities caring for vulnerable populations.

This study has several limitations. First, the pre–post design without a control group does not allow us to exclude potential testing effects or short-term learning bias; as the post-training assessment was conducted immediately after the session, observed improvements reflect immediate knowledge acquisition rather than long-term retention. Second, although the course was advertised to all school cafeterias and RSA collective catering within the institutional competence area and registration was open, selection bias cannot be excluded, as it could not be determined whether attendance was driven by personal motivation, organizational encouragement, or company obligation. Third, this study specifically evaluated only the ‘Knowledge’ component of the Knowledge, Attitudes, and Practices (KAP) model of food handlers, without assessing attitudes or observed practices. An integrative review by Zanin et al. [5] reported that there may be no translation of knowledge into attitudes or practices after training, and that satisfactory results in this triad are observed only when more advanced education and training techniques are used. Therefore, since knowledge was assessed through a structured questionnaire rather than direct observation of food handling practices, improvements in test scores may not necessarily translate into behavioral change in real-world settings. In addition, the questionnaire was developed based on regulatory requirements and training objectives but, given the observational pre–post nature of the study, was not subjected to formal validation or pilot testing prior to data collection. Longitudinal follow-up assessments are needed to evaluate the durability of training effects and to determine whether short-term improvements in knowledge translate into sustained competence over time.

In conclusion, this study assessed food safety knowledge and the effectiveness of a food safety training session among food handlers employed in school cafeterias and residential care facility catering services. Baseline food safety knowledge among collective catering staff was significantly associated with primary job role, highlighting potential differences between managers, food preparers, and food service workers. In particular, personnel mainly involved in meal distribution showed lower baseline knowledge levels, suggesting the need for greater attention to this professional group. Following the training session, immediate post-training knowledge improved across all professional categories, with a reduction in pre-existing disparities between roles. Nevertheless, persistent gaps in technically demanding areas highlight the need for continuous, role-tailored HACCP-based training. Ensuring that all personnel engaged in food distribution are adequately trained as food handlers remains a priority to protect vulnerable populations in institutional catering settings. Future studies should further investigate long-term knowledge retention and evaluate training effectiveness through larger samples and follow-up assessments.

## Figures and Tables

**Table 1:** Characteristics of food handlers and associations with positive test success before training session.

	All Food Handlers	Before Training Session			After Training Session		
		Test Passed Successfully ^#^		*p*-Value *	Test Passed Successfully ^#^		*p*-Value *
		No	Yes		No	Yes	
Total	168 (100)	115 (100)	53 (100)		41 (100)	125 (100)	
Gender							
Female	137 (82%)	95 (83%)	42 (79%)		35 (85%)	101 (81%)	
Male	30 (18%)	19 (17%)	11 (21%)	0.5	6 (15%)	23 (19%)	0.6
Age							
Median [range]	50 [24–64]	50 [25–63]	51 [24–64]	0.5	50 [31–63]	51 [24–64]	>0.9
Education							
Less than a high school diploma	67 (41%)	48 (43%)	19 (36%)		19 (49%)	48 (39%)	
High school diploma	77 (47%)	51 (46%)	26 (49%)		18 (46%)	58 (47%)	
Bachelor’s or Master’s degree	21 (13%)	13 (12%)	8 (15%)	0.6	2 (5.1%)	18 (15%)	0.2
Catering setting							
School cafeteria	93 (55%)	60 (52%)	33 (62%)		20 (49%)	73 (58%)	
RSA/Care canteen	71 (42%)	53 (46%)	18 (34%)		20 (49%)	49 (39%)	
Both	4 (2.4%)	2 (1.7%)	2 (3.8%)	0.2	1 (2.4%)	3 (2.4%)	0.6
Type of canteen							
Preparation and serving	101 (62%)	64 (57%)	37 (71%)		25 (64%)	76 (61%)	
Preparation only (cooking center)	19 (12%)	12 (11%)	7 (13%)		3 (7.7%)	16 (13%)	
Serving only (delivered meals)	44 (27%)	36 (32%)	8 (15%)	0.073	11 (28%)	32 (26%)	0.8
Job Work Main Responsibilities							
Food Preparer	90 (55%)	58 (51%)	32 (63%)		20 (50%)	70 (57%)	
Food service workers	56 (34%)	49 (43%)	7 (14%)		18 (45%)	37 (30%)	
Manager	19 (12%)	7 (6.1%)	12 (24%)	**<0.001**	2 (5.0%)	16 (13%)	0.2
Experience in Catering Sector							
Less than 1 year	10 (7.1%)	8 (8.5%)	2 (4.3%)		2 (5.9%)	8 (7.5%)	
1 to 3 years	11 (7.9%)	7 (7.4%)	4 (8.7%)		2 (5.9%)	9 (8.5%)	
4 to 6 years	13 (9.3%)	11 (12%)	2 (4.3%)		3 (8.8%)	10 (9.4%)	
More than 6 years	106 (76%)	68 (72%)	38 (83%)	0.4	27 (79%)	79 (75%)	>0.9
Experience in Collective Catering							
Less than 1 year	10 (6.7%)	6 (6.0%)	4 (8.2%)		1 (2.9%)	9 (7.8%)	
1 to 3 years	25 (17%)	15 (15%)	10 (20%)		9 (26%)	16 (14%)	
4 to 6 years	18 (12%)	13 (13%)	5 (10%)		4 (12%)	14 (12%)	
More than 6 years	96 (64%)	66 (66%)	30 (61%)	0.8	20 (59%)	76 (66%)	0.4
HACCP training course in the last 3 years							
No	68 (43%)	48 (44%)	20 (40%)		17 (44%)	50 (42%)	
Yes	92 (58%)	62 (56%)	30 (60%)	0.7	22 (56%)	69 (58%)	>0.9

Note: * *p*-value based on Fisher’s exact test for categorical variables and Wilcoxon test for continuous variables. ^#^ Correct answer of at least 11 out of 15 questions. Statistically significant *p*-value are reported in bold.

**Table 2:** Univariate and multivariate models of baseline knowledge test.

Variable	Univariate OR (95% CI)	*p*-Value	Multivariate OR (95% CI)	*p*-Value
Gender (Male vs. Female)	1.31 (0.56–2.96)	0.52		
Age (10-year increase)	0.98 (0.95–1.02)	0.26		
Education				
High school vs. Less than a middle school diploma	1.29 (0.63–2.65)	0.49	0.85 (0.37–1.96)	0.70
Degree vs. Less than a middle school diploma	1.55 (0.54–4.32)	0.40	0.76 (0.14–3.46)	0.73
Catering setting (School cafeteria vs. RSA)	1.62 (0.82–3.25)	0.17	2.92 (1.16–8.11)	**0.03**
Type of canteen				
Cooking center vs. Delivered meals	2.62 (0.77–8.92)	0.12		
Preparation and serving vs. Delivered meals	2.60 (1.14–6.55)	**0.03**		
Experience in catering sector (More than 6 years vs. Less)	1.82 (0.77–4.65)	0.19	2.07 (0.68–7.11)	0.22
Experience in collective catering (More than 6 years vs. Less)	0.81 (0.40–1.66)	0.57		
HACCP training course in the last 3 years (Yes vs. No)	1.16 (0.59–2.31)	0.67	0.48 (0.18–1.25)	0.14
Job work main responsibilities				
Food preparer vs. Food service	3.86 (1.65–10.22)	**0.003**	3.30 (1.10–11.10)	**0.04**
Manager vs. Food service	12.00 (3.69–43.60)	**<0.0001**	23.74 (4.16–168.5)	**0.0007**

Note: Statistically significant *p*-value are reported in bold.

## Data Availability

The original contributions presented in the study are included in the article/Supplementary Materials, further inquiries can be directed to the corresponding author.

## Supplementary Materials

The following supporting information can be downloaded at https://www.mdpi.com/article/10.3390/foods15132298/s1: Table S1: Questionnaire (knowledge section) administered before (pre-training) and after (post-training) the course. Table S2: Association between correct survey response at questions and food handlers job work main responsibilities. Table S3: Associations between food handler characteristics and sociodemographic data. Table S4: Distribution of knowledge scores before and after the educational intervention.
